# X-ray Computed Tomography Imaging of the Microstructure of Sand Particles Subjected to High Pressure One-Dimensional Compression

**DOI:** 10.3390/ma9110890

**Published:** 2016-11-03

**Authors:** Asheque al Mahbub, Asadul Haque

**Affiliations:** Department of Civil Engineering, Monash University, Victoria 3800, Australia; asheque.mahbub@monash.edu

**Keywords:** 3D X-ray CT imaging, 1-D compression, particle crushing, microstructure, sands

## Abstract

This paper presents the results of X-ray CT imaging of the microstructure of sand particles subjected to high pressure one-dimensional compression leading to particle crushing. A high resolution X-ray CT machine capable of in situ imaging was employed to capture images of the whole volume of a sand sample subjected to compressive stresses up to 79.3 MPa. Images of the whole sample obtained at different load stages were analysed using a commercial image processing software (Avizo) to reveal various microstructural properties, such as pore and particle volume distributions, spatial distribution of void ratios, relative breakage, and anisotropy of particles.

## 1. Introduction

Practical application of engineering mechanics on soils requires an in-depth understanding of their mechanical behaviour. In modelling soil behaviour, a soil mass has often been treated as a continuum, however, in reality it is composed of individual particles. The mechanical behaviour of soils (strength and deformation behaviour) is governed by the arrangement of soil particles (known as fabric) and the interparticle bonds, together they are known as soil structure [1]. In the case of reconstituted soils or granular soils, where the bond is negligible or even absent, its microstructure becomes basically the arrangements of particles and interparticle voids.

Although geotechnical problems deal with low stresses, there are cases where soils can be subjected to significantly high stresses, such as large earth dams, deep driven piles, and deep well shafts [2]. Under high stresses in one-dimensional compression, crushing of granular materials occurs, a phenomenon which was first described by Terzaghi and Peck [3]. Later, De Souza [4] linked particle crushing with the yield stress of granular soils. Development of a constitutive model for granular materials demands an in-depth understanding of the micro-structure and its evolution. Under low vertical stresses, grain-scale frictional slip, rotation and sliding accompanied by an insignificant particle crushing, contribute to the compaction of granular materials [5]. However, at high vertical stresses, a significant crushing of particles is known to take place which causes large deformations associated with tight grain packing and reduction of void ratios. Particle crushing is strongly associated with the tensile strength of individual grains [6]. However, some other factors such as the particle morphology and minerology, particle distribution, contacts, orientations, and void ratio which contribute to the fabric of granular materials, significantly influence the complex micro-mechanical behaviour of granular materials. Many researchers have studied the crushing and yielding behaviour of granular soils subjected to one-dimensional compression loading [4,7,8,9,10,11,12,13,14,15]. 

De Souza [4] and Hendron [7] reported that the yield stress increases with the increase of initial density, and decreases with the increase of particle angularity. Hendron [7] observed that the effect of initial void ratio, which is significant at low stresses, diminishes at higher stresses. Analysing initial and crushed particle size distributions, Hardin [8] suggested valuable equations for estimating the degree of particle crushing. Fractal particle distribution from successive crushing of soil particles gives a linear distribution on a double logarithmic graph [9,10,11]. Results of investigation by Hagerty et al. [12] agreed well with the findings of earlier researchers, in particular, a significant increase of particle crushing was observed with the increase of median particle size. The change of particle sizes with stresses affects many important properties of sands, such as the yield stress, compressibility, susceptibility to erosion, shear strength, and hydraulic conductivity. The effect of particle size distributions on the grain crushing under 1D compression was studied by Nakata et al. [13] and Altuhafi and Coop [14]. They concluded that a uniformly graded sand shows marked yielding compared to a well graded sand and they related such differences with the nature of the microscopic crushing of the particles. Nakata et al. [13] pointed out that after reaching the yield stress, a sudden increase in particle crushing was obvious and it took place mostly within the smaller size particles. In order to study crushing behaviour under 1D compression, Nakata et al. [13] conducted a series of tests, each of which was terminated at a defined stress level, and sieve analysis was performed for each case to determine particle size distributions. Altuhafi and Coop [14] concluded more breakage occurs in samples with higher initial void ratios.

Past studies on the 1D compression of soils heavily relied on invasive tests, which tend to disturb the fabric of soils. More importantly, it was not possible to examine non-destructively the micro-structural changes of soils subjected to an incremental stress regime. In addition, particles for observation had to be chosen randomly and in limited number which greatly influences the accuracy of the outcome. Due to these limitations, researchers have been relying on numerical tools (such as the Discrete Element Method (DEM)) to investigate the material behaviour under different loading conditions [15,16,17,18,19]. However, the DEM uses idealized digital particles which do not truly represent the actual particle size distributions in many cases. Therefore, to make the DEM more close to the reality, efforts have to be made to precisely characterize the micro-structure of soils.

High resolutions X-ray computed tomography (X-ray CT) is a robust and non-destructive imaging technique, which can be used to overcome many difficulties through 3D visualization and quantification of many parameters closely describing the fabric of granular soils. Some researchers [20,21,22] used this technology for the quantification of fundamental particle characteristics, such as particle morphology, contact etc., while others [23,24,25,26] used it for the quantification of the microstructural evolution of granular soils subjected to biaxial [25] and triaxial loading [26], where the focus was mainly on the development of shear bands. However, with the advancement of laboratory-scale X-ray CT technology and image segmentation techniques [27,28], it is now possible to conduct investigation at grain-scale level. For example, Fonseca et al. [23] used X-ray CT to capture images of intact sample at a very high resolution (voxel size = 5 microns) and compared them with the gradations of reconstituted samples. However, in this study, only sub-volumes instead of the whole sample were scanned at the end of triaxial tests. Recently, Zhao et al. [29] conducted in situ imaging of a single sand grain under very high compression loading with the objective of understanding the particle fracture mechanisms.

This paper investigates the 1D compression behaviour of uniformly graded sands using a high resolution 3D X-ray CT machine. In situ imaging of the whole sample was performed at the end of each load, and images were post-processed and analysed to study the evolution of microstructure of a sand sample subjected to a very high 1D compression loading leading to sand particle crushing.

## 2. Laboratory Investigation

### 2.1. Materials

**Figure 1:**
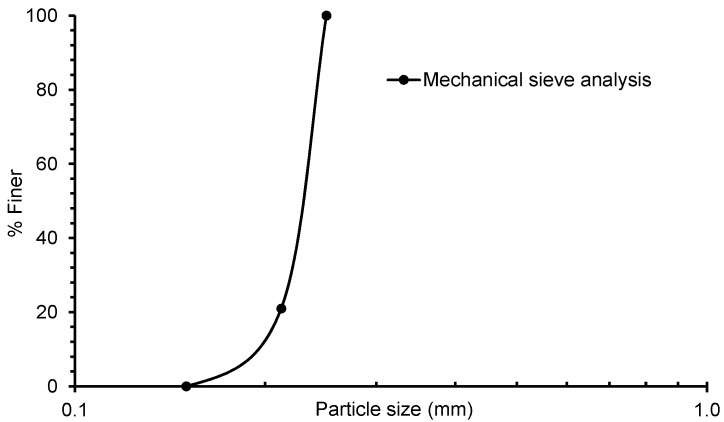
Initial particle size distributions from mechanical sieve analysis.

In this study, a commercially available washed sand sample was sourced from a natural deposit site located at Gippsland in Victoria, Australia. The sand particles are brown in colour and sub-angular in shape. First, the sand particles were dried in an oven for 24 h at 105 ± 0.5 °C. Second, the particles were sieved through 250, 212, and 150 microns sieves. In this study, sand particles passed through the 250 microns and were retained on the 212 and 150 microns sieves used. The particle size distribution plot is shown in Figure 1. The values of the coefficient of uniformity (C_u_) and the coefficient of curvature (C_c_) were 1.24 and 1.06 respectively, which classified the sand to be uniformly graded as per the unified soil classification system. The mean diameter (D_50_) of the sand particles was 230 microns. The selection of this size is important to achieve an acceptable representation of sand particles within the volume [30] including the minimum height to diameter ratio of the sample to be tested under 1D compression with a full field of view imaging. X-ray diffraction test showed that the sand particles were comprised of quartz minerals. The specific gravity of sand particles was measured three times using a Multipycnometer (Quantachrome Instruments, Boynton Beach, FL, USA), which produced an average value of 2.68.

### 2.2. Experimental Setup

**Figure 2:**
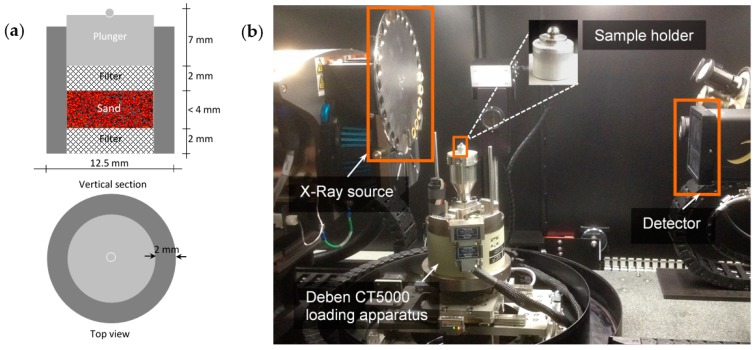
(**a**) Sample holder setup; (**b**) Test setup within X-ray computed tomography (X-ray CT) machine.

A new 1D compression apparatus was designed in this study. It comprises a 2-mm thick walled aluminium cylinder of 8.5 mm internal diameter and 10 mm height, two 2-mm thick bronze filters and a 6-mm height stainless steel plunger with a 1-mm diameter stainless steel ball. The aluminium cell was selected due to its low X-ray absorption capacity and the bronze filters for the dissipation of pore pressure under loading. A typical setup of the apparatus is shown in Figure 2a. A sliding fit of filters and plunger was ensured. It can hold samples of height up to 4 mm thus ensuring specimen minimum diameter to height ratio of 2.5 [31]. The sand particles were placed in the compression apparatus from a height of about 10 mm. The mass of the particles was recorded after it reached the target height of 3.5 mm, which corresponded to an initial bulk density of 1.62 g/cm^3^ (20,882 particles).

The 5 kN compressive load capacity load-stage (CT5000, Deben, Suffolk, IP30 9QS, UK) was used in this study. Figure 2b shows the image of the interior of the X-ray CT machine with the load-stage and 1D compression apparatus. It is to be noted that the clearance between the top and the bottom platens of the load-stage is 15 mm, which restricted the height of the sample to be tested in situ. The bottom platen of the load-stage moves upward and compresses the sample against the top reaction platen. The load-stage and data acquisition system are controlled by the MICROTEST software (V6.13) developed by Deben (Suffolk, UK) [32]. The system has a wide range of control functions (such as data acquisition interval, loading rate) and a continuous display window for plotting variations of a selected group of variables (such as load vs. deformation, deformation vs. time).

In the test, an initial load of 100 N was applied. Thereafter, the loads were doubled for the next five consecutive increments until they reached 3200 N. The final load after 3200 N was 4500 N due to the load capacity of the stage (5000 N). The vertical compressive stresses corresponding to the load cases were 1.8, 3.5, 7.0, 14.1, 28.2, 56.4, and 79.3 MPa. A target compressive load was achieved through the upward movement of the bottom platen at a rate of 0.1 mm/min. Once the load had reached a target value, the sample was allowed to undergo complete deformation under the constant load, which was monitored by reading axial deformation with time. It was found that about 30 to 60 min time was required to complete the immediate compression of the sample (i.e., the deformation-time plot reached an asymptotic value), where a higher time corresponded to a higher load. During the imaging, the movement of the bottom platen was paused and the final stress value at the end of the imaging was recorded, which showed less than 5% stress relaxation. At the completion of imaging, the next level of load was applied and the whole process was repeated.

### 2.3. Image Acquisition and Processing

An ultra-high resolution (0.7 microns, Zeiss Xradia XRM520Versa) X-ray Microscopy Facility (Xradia, Pleasanton, CA, USA) for Imaging Geo-materials (XMFIG) was used for image acquisition in this study. The XMFIG was established through an Australian Research Council Linkage Infrastructure and Equipment Fund (LE130100006) and equipped with various in situ imaging capabilities (such as unconfined, triaxial and 1D compression load stages).

Image projections were acquired by rotating the load-stage 360° around its vertical axis. The frame size of the projections was 1024 × 1024 pixels. The scanning parameters used for all the scans are given in Table 1.

In this study, a total of 801 projections with a pixel size of 14.28 microns (≈0.062D_50_) was taken, which took about half an hour. The 2D projections were reconstructed to 3D volumes using XRM Reconstructor software (Cone Beam-10, Xradia, Pleasanton, CA, USA) [33]. The full volume of the bounding box that contained the initial cylindrical sample was 600 × 600 × 225 voxels.

The reconstructed images were post-processed using a commercially available image processing software Avizo (V9.1.1, FEI, Hillsboro, OR, USA) [34]. Images of sand particles were cropped from the whole apparatus assembly followed by removal of noises using appropriate filters. Subsequently, the solid and void phases of the image were segmented and sand particles were separated and labelled for various quantitative analyses.

**Figure 3:**
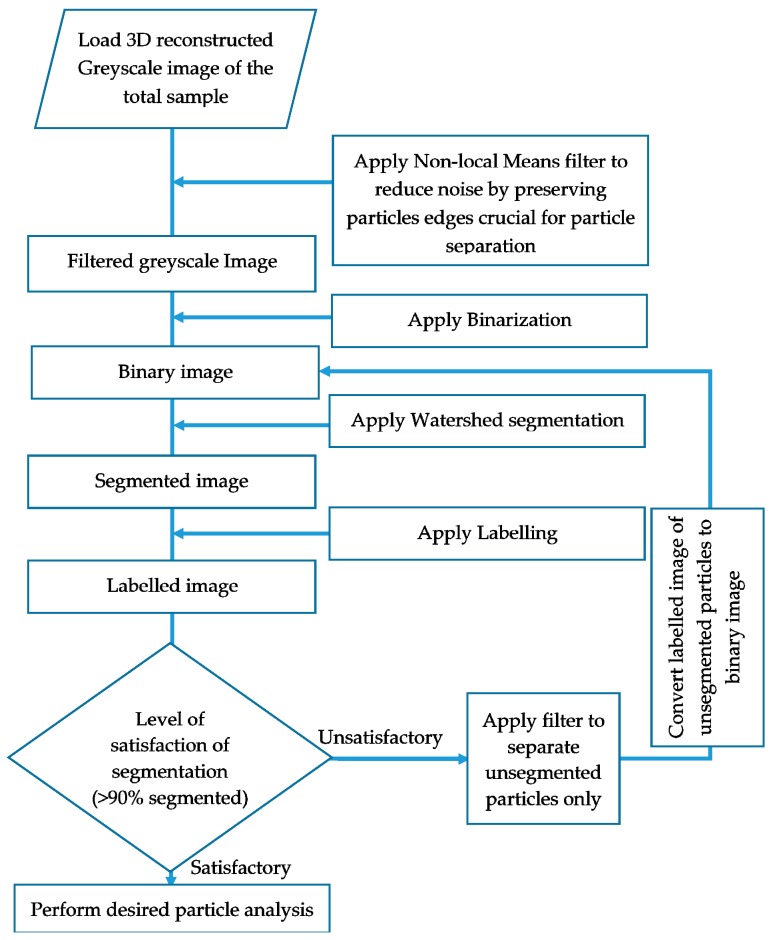
Flow chart depicting all operations of image processing in this study.

For noise reduction, the non-local means filter (FEI, Hillsboro, OR, USA) was used in this study. Despite being GPU accelerated, the runtime of this filter was very high compared to other filters such as the 3D median filter. However, the non-local filter was found to be highly effective in reducing noises while preserving the edges of particles which is critical for particle separation. After filtering, the greyscale image was transformed to binary image by applying the interactive thresholding module which prompts the user to set the grey level intervals manually with a visual feedback. As a preliminary identification of intensity ranges separating solids from voids, an intensity range partitioning tool was used which can automatically guess thresholds separating different densities of materials. Then by manually adjusting this preliminary threshold range in the interactive thresholding, voxels were defined as either solid (sand particle) or void by trial and error based on visual assessment (human judgement) [35] of their intensities. However, to perform grain scale analysis, further segmentation was required to separate sand particles from each other. The morphological watershed algorithm module was applied to perform this task of separating individual sand particles. The procedure consisted of: (a) calculating the Euclidian distance map (EDM) on the solid phase of the binary image by applying fast yet accurate approximation based Chamfer metric considering 26-neighbourhood when propagating the distance value; (b) identifying the local maxima of the EDM with contrast value of 1; (c) labelling the local maxima as markers; and (d) applying marker based watershed with 3D interpretation and 26-neighbourhood connectivity. In every load case, three to four iterations of watershed, depending on the extent of connected voxels resulting from increased loading, were found necessary to achieve the desired level (>90% particles separation) of segmentation. To avoid over segmentation, a subsequent run of the algorithm was conducted for only the particles which could not be separated in the previous instance. The whole image processing method applied in this study is depicted briefly in the flowchart shown in Figure 3.

## 3. Results and Discussions

### 3.1. Void Ratio vs. Logarithm of Vertical Stress (e-logσ’_v_) Plot

**Figure 4:**
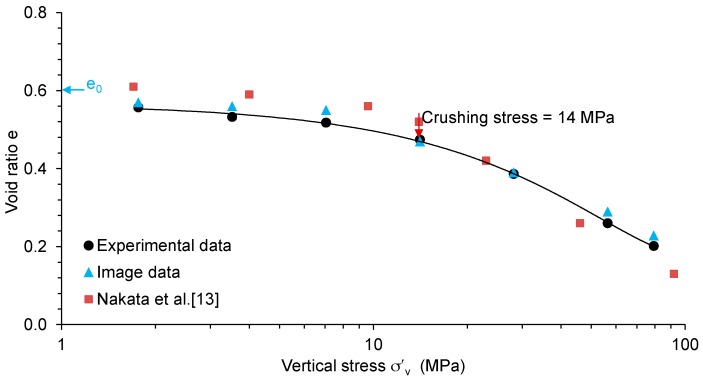
e-logσ’_v_ plot for the uniformly graded sand sample.

The experimental and physical measurements of the sand sample were utilised to calculate the void ratio at each load cases. The variations of void ratio with the logarithm of vertical stress (e-logσ’_v_) plot for the sand sample tested in this study is presented in Figure 4. The initial void ratio (e_o_) of the sample was 0.60.

The e-logσ’_v_ plot shows a gradual change of slope up to 7 MPa of vertical stress followed by a significant change of slope, which could be due to the crushing of particles. In this study, the crushing stress or yield stress is defined as the stress corresponding to the intersection of two straight parts of the e-logσ’_v_ curve [36,37]. The yield stress for the uniformly graded sands tested under 1D compression was found to be 14 MPa which is denoted by the arrow sign in the above figure. The observed value is similar to that reported by Nakata et al. [13] for uniformly graded quartz sand of relatively larger particle sizes but of almost similar uniformity coefficient (1.1 vs. 1.24 of this study) and initial void ratio of 0.6 ± 0.03. This indicates that the small compression apparatus designed in this study is capable of producing an acceptable result when compared with the result obtained from the relatively large apparatus (50 mm diameter and 10 mm height) of Nakata et al. [13].

**Figure 5:**
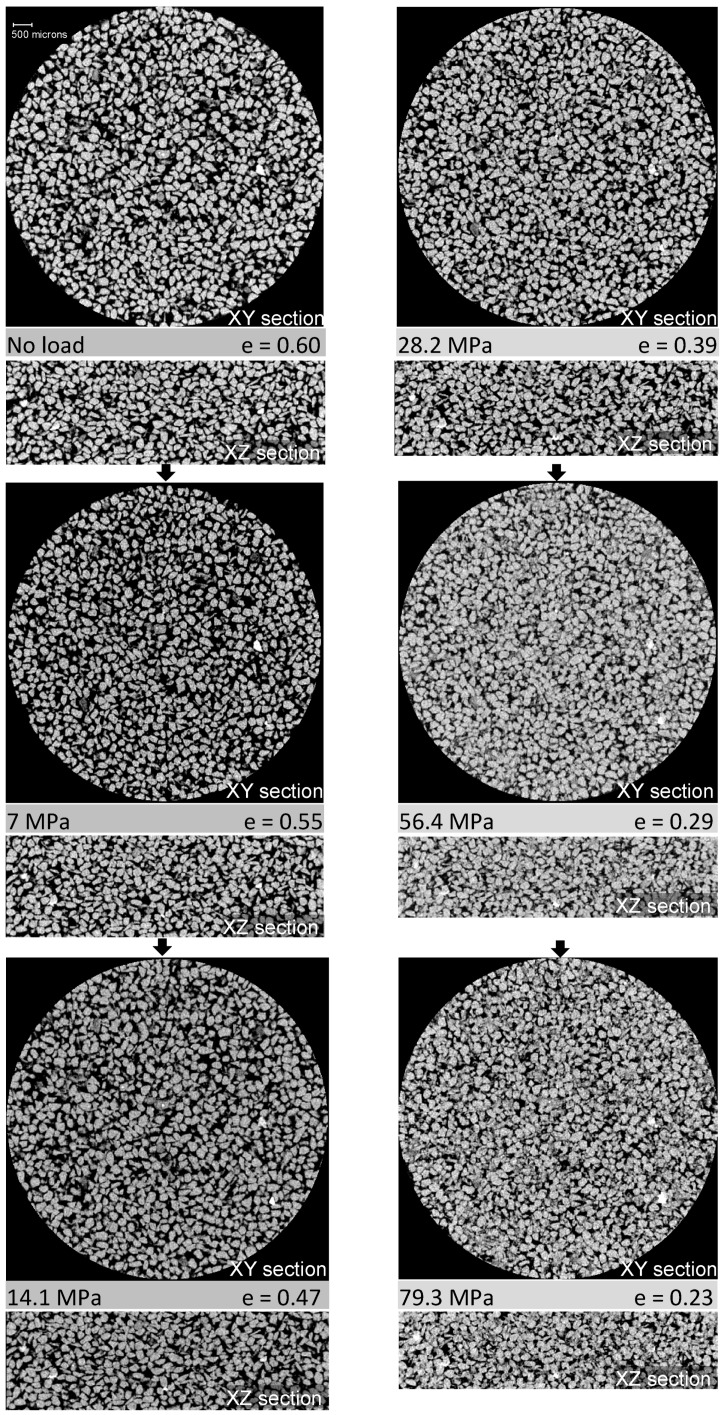
Horizontal and vertical image sections through the centre of the sample at different vertical stresses.

The e-logσ’_v_ plot also shows that the decrease of void ratio in the post-yield stresses is higher, indicating a higher degree of particle crushing [9]. The slope of the e-logσ’_v_ curve in the post-yield region is reasonably in agreement with that of Nakata et al. [13]. In order to explore the capability of 3D X-ray CT imaging, only the greyscale images of vertical and horizontal sections through the centre of the whole sample under six load cases including the initial state are presented in Figure 5. It is evident from these images that with increased stresses, especially after the yield stress (14 MPa), a significant collapse of voids and crushing of particles were encountered. At the maximum vertical stress of 79.3 MPa, the void ratio was observed to decrease to 0.21, which is one-third of the initial value (0.60). A void ratio close to 0.19 was reported by Nakata et al. [13] for the uniformly graded sand tested under a similar vertical stress.

### 3.2. Evolution of Microstructure

#### 3.2.1. Void Size Distributions with Vertical Stresses

**Figure 6:**
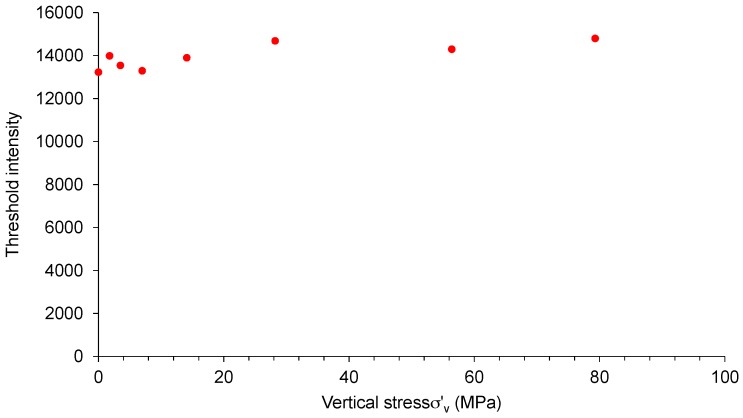
Threshold intensity values of grey scale images for different vertical stresses.

The images acquired for each load case were thresholded to obtain the volume of solid and void phases. Subsequently, the void ratios were calculated for each load cases and plotted together with the experimentally obtained values (Figure 4). It can be seen that the void ratios calculated from the image analysis using the threshold intensities as shown in Figure 6 are in good agreement with the experimental data. An increasing trend of threshold intensity values as shown in Figure 6 could be associated with the increase of the fraction of fines and the bulk density of the sand sample subjected to increased compressive loads.

**Figure 7:**
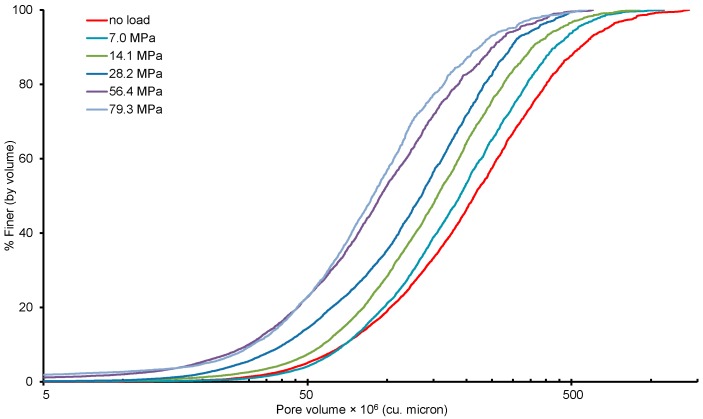
Pore volume distribution of samples tested under different vertical stresses.

The void volume obtained by the thresholding was further processed using the watershed algorithm to obtain the pore volume distributions. Figure 7 presents the distribution of pore volumes with increase in vertical stresses. Unlike particles, pores are interconnected and often form large volumes based on 26-neighbourhood connectivity. As anticipated, large pores were observed to reduce to small size pores with the increase of vertical stresses. Interestingly, not much difference between the pore size distribution plots for the final two load cases was observed, which is in-line with the relatively small change of void ratios experienced under these stresses (Figure 4).

#### 3.2.2. Void Ratio Distributions

**Figure 8:**
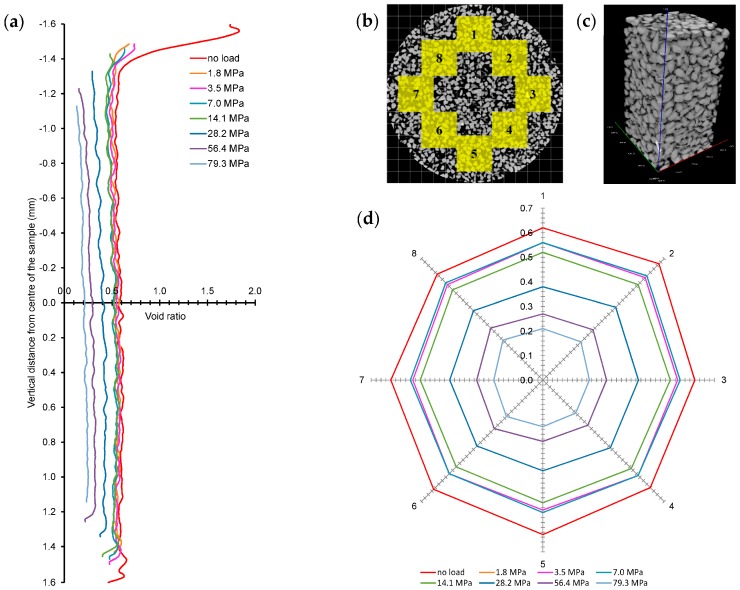
(**a**) Change of void ratios along the height of the sample with increased vertical stresses; (**b**) Locations of eight sub-volumes selected for spatial analysis; (**c**) 3D image of a sub-volume; (**d**) Spatial distribution of void ratios of sub-volumes with increased vertical stresses.

The 3D reconstructed slices of 8.5 mm × 8.5 mm × 0.14 mm (600 × 600 × 1 voxels) size were analysed to determine the change of void ratios with the height of the sample for all the load cases (Figure 8a). A shift of the plot to the left indicates a reduction of void ratio under the effect of vertical stress. It can be seen that the void ratios almost remained unchanged within the height of the sample for all the load cases except for the initial case where the top 100 microns of the sample had a higher void ratio due to the uneven surface characteristics. Moreover, the change of void ratios with load cases up to the yield stress of 14 MPa were insignificant (0.6 at no load to 0.57 at 1.8 MPa to 0.55 at 7.0 MPa) compared to the values observed for higher stresses exceeding 14.1 MPa where crushing of particles was encountered. The crushing of particles resulted in a more uniform distribution of void ratios along the height of the sample (Figure 8a).

The spatial distribution of void ratios with the increase of vertical stresses was further investigated by selecting eight sub-volumes or representative elementary volume (REV) of 1.7 mm × 1.7 mm × 3.2 mm (side length > 7D_50_) [35] with a total voxels count of 3,175,873 (Figure 8b,c). It can be seen that the initial value of the void ratios for the REVs compared reasonably well with the initial void ratio of the whole volume (e_o_ = 0.60). A wagon wheel plot of the void ratios calculated from the image analysis for the REVs under different load cases is shown in Figure 8d. As expected the void ratio of the REVs decreases with the increase in pressure. Interestingly, the void ratio of the REVs under a given pressure is observed to be almost the same. Moreover, these values were very close to the values obtained along the full height of the sample (Figure 8a), indicating image analysis of a properly selected REV could produce meaningful outcomes of pressure-void ratio variations under very high pressure.

#### 3.2.3. Particle Size Distributions

##### Initial Distributions

**Figure 9:**
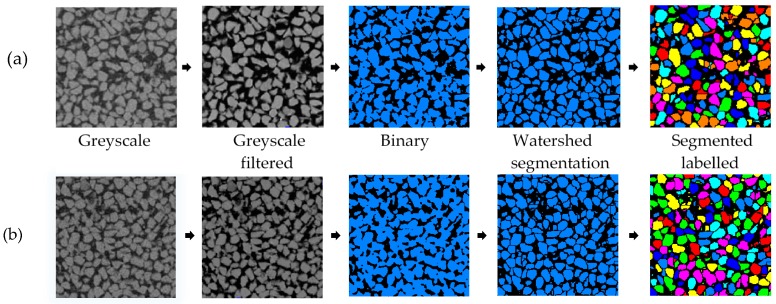
Examples of image processing depicting particle separation and identification for (**a**) no load; and (**b**) 14.1 MPa.

**Figure 10:**
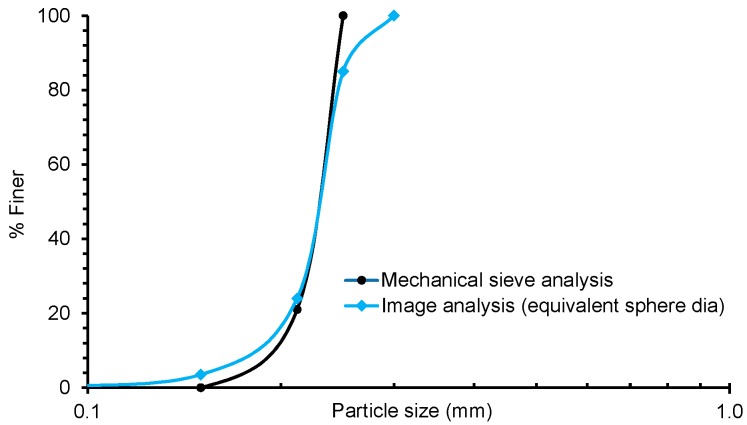
Initial grading obtained from mechanical sieving and image data.

3D X-ray CT image of the initial sample was post-processed and separated using the procedure mentioned in Figure 3. Two examples, one for no load and another for 14.1 MPa, of the process of separation are presented in Figure 9. The volume of particles obtained from the label analysis was used to calculate the equivalent sphere diameter of particles, which was then compared with the particle size distributions obtained from the sieve analysis (Figure 10). It is understood that different size descriptors (e.g., Feret diameter, equivalent sphere diameter) will have different degrees of success [38] when correlating with the particle size distribution curve obtained from the mechanical sieve analysis. In this study, the equivalent sphere diameter of the 3D volume of particles was considered due to its wide application in laser particle size analysis. It can be seen that the particle size distribution curve obtained from the image analysis compares well with the sieve analysis curve for the uniformly graded sand particles tested in this study. Existence of insignificant percentage (<4%) of particles smaller than 150 microns (Figure 10) could be due to the tolerances permitted in the average opening of the testing sieves and abrasion of particles during sieving, which are usually less than 5% of the total sample [39].

Above 80% finer, the distribution obtained from the image analysis shows presence of particles of equivalent sphere diameters as large as 300 microns or even more. This value is some 20% higher than the opening size of the largest mesh (250 microns) used in this study. The only possible reason for relatively larger volume particles to pass through the smaller sieve size could be related to the large value of particle size anisotropy (which is discussed in a later section of this paper), and orientation. A further verification using the sand particles volume obtained from the image analysis confirms that the bulk density of the sample is in good agreement with the measured initial bulk density (Table 2).

##### Load-Dependent Distributions

**Figure 11:**
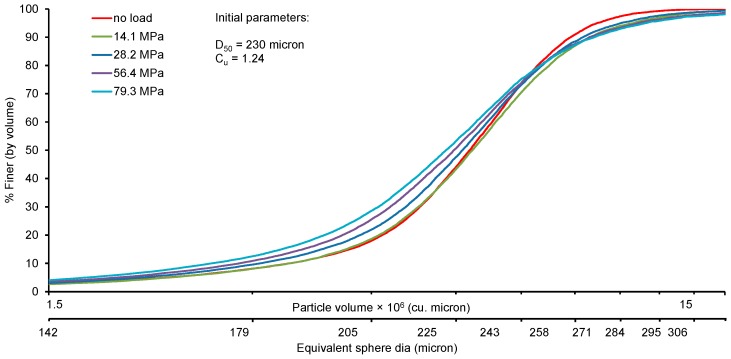
Evolution of particle size distribution from crushing at different loads.

Figure 11 shows the evolution of the particle size distribution with vertical stresses up to 79.3 MPa. For each load case, the images were analysed to obtain the particle size distributions (PSD). As there was very little change of particle size distributions up to a yield stress of 14 MPa, for the sake of clarity of the presentation only the evolution of PSD for stresses higher than the yield stress are presented. Both the 3D volume of particles and their equivalent sphere diameters were considered for plotting the PSD.

**Figure 12:**
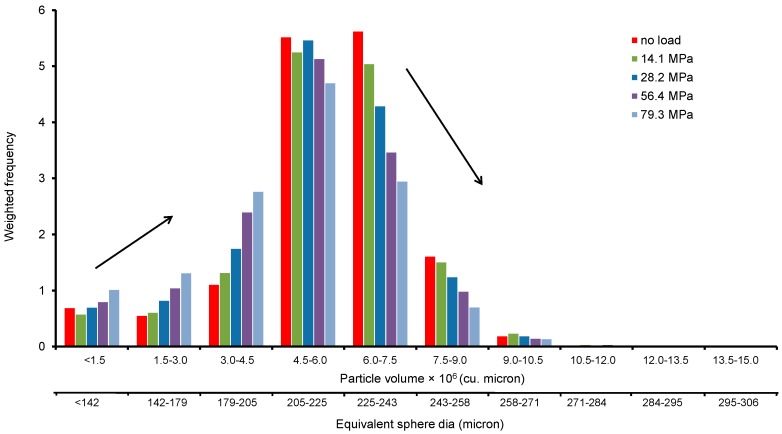
Particle size frequency distribution.

The nature of the gradual upward shifting of the PSD curves in Figure 11 from no load condition to subsequent higher loads indicates generation of smaller size particles due to particle crushing. The development of a pivot point around 250 microns equivalent sphere diameter and an upward sifting of PSD below this size indicate particle crushing predominantly at the pivot point and below. Similar observations were reported by Nakata et al. [13]. However, this study finds relatively less breakage of particles in terms of increase of fines. The reason might be due to the smaller mean particle size (D_50_ = 230 microns) in this study compared to a much higher value (D_50_ = 1550 microns) of Nakata et al. [13], which poses a higher potential to breakage [8]. On the other hand, particles above the 250 microns size showed an unexpected downward shift of the PSD curve compared to the no load curve. This could be related to the large equivalent diameter of the small number of unseparated particles in the processed image. However, their influence on explaining the particle crushing behaviour of the sand sample can be considered relatively small (Figure 12).

In order to understand the evolution of PSD with increased loading, the frequency of a defined range of particles and their corresponding volume fraction with respect to the total volume of the sample were determined. A weighted frequency is calculated by multiplying the volume fraction of a range of particle size with its frequency i.e., weighted frequency = number of particles (%) × volume fraction. Figure 12 shows the change of weighted frequency of various particle ranges with increased stresses. It is evident from the plot that crushing of particles occurred predominantly within the 205 to 258 microns size. In particular, particles of size range between 225 and 243 microns showed a significant drop of weighted frequency with loading (5.5 for no load to 3 for 79.3 MPa), indicating a relatively high crushing of particles with sizes close to the mean particle diameter (D_50_ = 230 microns). Interestingly, particle sizes close to 0.93D_50_ diameter (i.e., 205–225 microns) showed a very small change of the weighted frequency (5.5 for no load vs. 5 for 56.4 MPa) except for the 79.3 MPa, where a weighted frequency value of approximately 4.5 associated with a higher degree of crushing was observed. As expected, the weighted frequency of particles below 205 microns size was observed to increase with the increase of stress. This indicates the formation of new particles of smaller sizes at the expense of crushing of larger particles (>D_50_ size) with increased loading. This was also reflected in the rapid reduction of the effective particle diameter (D_10_) with increased loading (Figure 11).

#### 3.2.4. Particle Breakage

**Figure 13:**
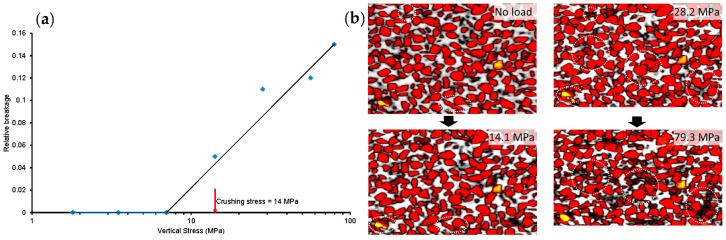
Particle breakage under different vertical stresses: (**a**) relative breakage; (**b**) images showing breakage of particles.

The relative breakage parameter (B_r_), which is defined by Hardin [8] as B_t_/B_p_ where B_t_ = total breakage and B_p_ = breakage potential, was calculated for all load cases (Figure 13a). It can be seen that the values of B_r_ for stresses below the crushing stress (14 MPa) are insignificant (B_r_ ≈ 0). In the post-crushing stress range, a linear variation between B_r_ and stress can be approximated, which is in good agreement with Coop and Lee [40]. This linear variation of B_r_ can be explained with the help of the gradual change of the slope of the e-logσ’_v_ plot in the post-crush region (Figure 4). It is believed that if the applied stress is significantly higher than the presently used maximum stress (79.3 MPa), the value of B_r_ may reach an asymptotic value indicating no further breakage of particles. Figure 13b illustrates the breakage of particles at high stresses relative to no load condition. The breakage of particles under high stresses is marked by circles. The different colours of particles in Figure 13b indicate different intensity values.

#### 3.2.5. Particle Size Anisotropy

Particle size anisotropy is defined as 1 minus the ratio of the smallest to the largest eigenvalue of the covariance matrix [34]. Anisotropy measures a particle’s deviation from a spherical shape, with a value of 1 indicating highly non-spherical particle and a value of zero indicating a fully spherical particle.

**Figure 14:**
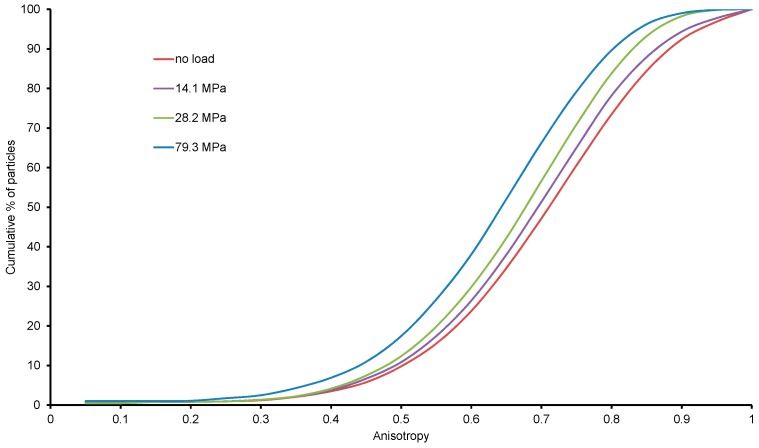
Anisotropy distributions of particles with vertical stresses.

**Figure 15:**
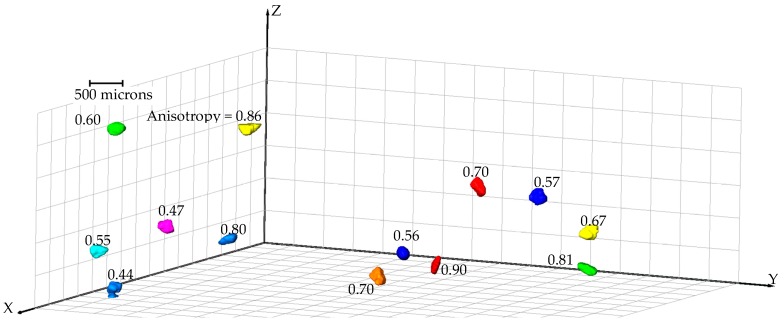
Variation of anisotropy of particles with equal volumes (6.3 × 10^6^ cubic microns).

Size anisotropy for all the particles under no load condition was determined from the labelled images and their distributions are plotted in Figure 14. It is clear that more than 80% of particles had anisotropy values greater than 0.6. Therefore, it is likely, during mechanical sieving, that a particle with a given volume may pass a square mesh size which is smaller than the equivalent sphere diameter at a suitable orientation as opposed to a particle with the same volume but with a lower anisotropy value. This phenomenon is illustrated in Figure 15, where thirteen particles of equal volume (6.3 × 10^6^ cubic microns) are shown with varying anisotropy values (0.44 to 0.90). Due to the relatively large long-axis dimension of particles associated with high anisotropy values, these particles will have a greater chance of passing through sieve sizes smaller than their equivalent diameters.

With the increase of load, particles undergo more grinding and breakage leading to particles more spherical in shape. This results in decrease of anisotropy (particles become more spherical) with an increase of loading which has been depicted from the inward shift of the anisotropy distribution curves (Figure 14).

**Figure 16:**
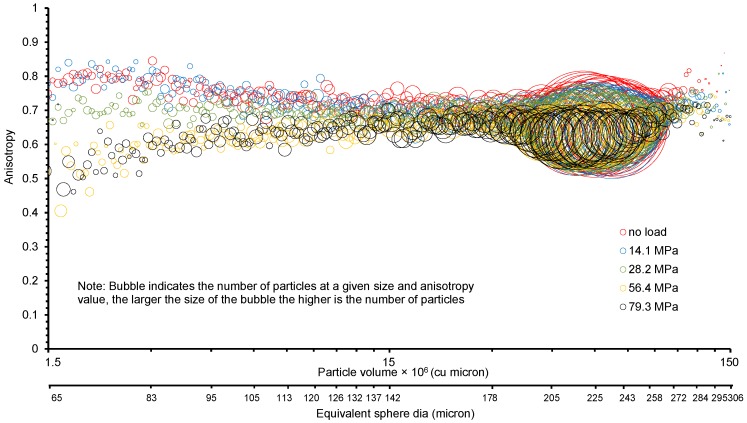
Particle anisotropy and frequency distributions with vertical stresses.

Figure 16 shows a bubble plot representing the frequency distribution of particles of various sizes and their anisotropy with different vertical stresses. It is observed that with the increasing load, anisotropy, in general, decreases and the bubble size representing the frequency of particles increases indicating formation of smaller size particles due to crushing.

## 4. Conclusions

High resolution X-ray CT in situ imaging of microstructure of sand particles subjected to high pressure one dimensional compression leading to particle crushing was conducted. The images taken at different load stages were analysed to capture the micro-structural characteristics including the void and particles volume distributions, change of void ratios, range of particles undergoing crushing, and distribution of particle size anisotropy. The outcomes of this study are summarised below.

- The small-scale 1D compression apparatus setup designed for in situ X-ray CT imaging was found to produce comparable results for e-logσ’_v_ variations including the yield strength obtained from the lab-scale 1D compression test performed by Nakata et al. [13] on uniformly graded sand particles of similar uniformity coefficients. The void ratios calculated for each load cases from the image analysis were in good agreement with the experimental data, including the initial bulk density of the sand particles tested.
- The increase of loads resulted in decreased global void ratios and pore volume sizes. Moreover, the void ratio variations along the height of the sample showed a gradual decrease until the yield stress and thereafter, a significant decrease instigated by marked particles crushing. With the increase of stresses, the sinusoidal variations of void ratios encountered along the height of the sample under low stresses were observed to diminish. A more uniform change of void ratio of sub-volumes (REV) located at peripheral positions was also observed.
- The initial particle size distributions of sand particles obtained from mechanical sieve analysis and that obtained from the image analysis using an equivalent sphere diameter were in reasonably good agreement. The evolution of particle size distributions resulting from the crushing of particles under incremental stresses could be captured using the non-destructive X-ray CT in situ imaging. Moreover, the specific size group of particles predominantly subjected to crushing or forming under different stresses could be identified. With increased stresses, the creation of more fine particles associated with reduced values of size anisotropy was evident.
- The crushing of sand particles in the pre-yield stress region was insignificant as supported by the near zero values of the relative breakage parameter. The value of the relative breakage parameter was found to increase at the onset of yielding and thereafter, a linear variation with logarithm of vertical pressure could be approximated.

The non-destructive X-ray CT imaging of micro-structure and analysis of image data for sand particles subjected to high pressure one dimensional compression are believed to add significant insight into the development of robust soil models using the discrete element modelling technique (DEM), where the particles can be modelled as crushable with the capacity of accommodating microstructural evolution. The outcomes will serve as the basis for future X-ray CT investigation, which are currently underway, on cemented sand particles subjected to one dimensional compression.

## Figures and Tables

**Table 1:** Scanning parameters and their values.

Parameter	Value/Description
Source to sample distance	85 mm
Detector to sample distance	322 mm
Voltage	140 keV
Power	10 W
Exposure time	2.5 s
Camera binning	2
Lens	Macro (0.4×)

**Table 2:** Calibration of image data with physical measurement.

Mass-Volume-Density Relationships	Image Analysis
Initial bulk density (*ρ*_i_) = 1.62 g/cm^3^	Total volume of sand particles (V_s_) = 0.09 cm^3^
	Specific gravity of sand particles (G_s_) = 2.68
	Mass of sand particles (M_c_) = V_s_G_s_*ρ*_w_ = 0.24 gm
	Bulk volume (V_b_) = 0.15 cm^3^
	Bulk density = M_s_/V_b_ = 1.60 ≈ 1.62 g/cm^3^
